# Understanding gender dynamics in mHealth interventions can enhance the sustainability of benefits of digital technology for maternal healthcare in rural Nigeria

**DOI:** 10.3389/fgwh.2022.1002970

**Published:** 2022-09-06

**Authors:** Ogochukwu Udenigwe, Friday E. Okonofua, Lorretta F. C. Ntoimo, Sanni Yaya

**Affiliations:** ^1^School of International Development and Global Studies, Faculty of Social Sciences, University of Ottawa, Ottawa, ON, Canada; ^2^Women's Health and Action Research Centre, Benin City, Edo, Nigeria; ^3^Centre of Excellence in Reproductive Health Innovation, University of Benin, Benin City, Nigeria; ^4^Department of Demography and Social Statistics, Federal University Oye-Ekiti, Oye-Ekiti, Ekiti, Nigeria; ^5^The George Institute for Global Health, Imperial College London, London, United Kingdom

**Keywords:** mHealth, maternal health, gender dynamics, digital health technologies, engaging men

## Abstract

**Introduction:**

Nigeria faces enormous challenges to meet the growing demands for maternal healthcare. This has necessitated the need for digital technologies such as mobile health, to supplement existing maternal healthcare services. However, mobile health programs are tempered with gender blind spots that continue to push women and girls to the margins of society. Failure to address underlying gender inequalities and unintended consequences of mobile health programs limits its benefits and ultimately its sustainability. The importance of understanding existing gender dynamics in mobile health interventions for maternal health cannot be overstated.

**Objective:**

This study explores the gender dimensions of Text4Life, a mobile health intervention for maternal healthcare in Edo State, Nigeria by capturing the unique perspectives of women who are the primary beneficiaries, their spouses who are all men, and community leaders who oversaw the implementation and delivery of the intervention.

**Method:**

This qualitative study used criterion-based purposive sampling to recruit a total of 66 participants: 39 women, 25 men, and two ward development committee chairpersons. Data collection involved 8 age and sex desegregated focus group discussions with women and men and in-depth interviews with ward development committee chairpersons in English or Pidgin English. Translated and transcribed data were exported to NVivo 1.6 and data analysis followed a conventional approach to thematic analysis.

**Results:**

Women had some of the necessary resources to participate in the Text4Life program, but they were generally insufficient thereby derailing their participation. The program enhanced women's status and decision-making capacity but with men positioned as heads of households and major decision-makers in maternal healthcare, there remained the possibility of deprioritizing maternal healthcare. Finally, while Text4Life prioritized women's safety in various contexts, it entrenched systems of power that allow men's control over women's reproductive lives.

**Conclusion:**

As communities across sub-Saharan Africa continue to leverage the use of mHealth for maternal health, this study provides insights into the gender implications of women's use of mHealth technologies. While mHealth programs are helpful to women in many ways, they are not enough on their own to undo entrenched systems of power through which men control women's access to resources and their reproductive and social lives.

## Introduction

An increasing number of interventions targeted toward social and behavior change are recognizing health behaviors as embedded in social and structural factors such as gender norms and unequal access to resources. This has led to an increased call for gender integration into health interventions, particularly digital health innovations which are at the forefront of transforming maternal healthcare delivery (1). Digital health, more specifically mobile health or mHealth is defined as the use of mobile devices to support medical and public health practices (2). Mobile devices such as mobile phones, patient monitoring devices, digital assistants and wireless devices support a broad spectrum of mHealth programs for maternal health including telephone helplines, text message appointment reminders, mobile telehealth, and mobile patient electronic information.

Mobile health primarily serves to improve access to healthcare by increasing the speed of access and reducing cost, particularly in countries where there is inequity in the distribution of healthcare. For instance, in Nigeria where the global burden of maternal mortality is dire, there is a remarkable gap in access to skilled healthcare personnel based on women's socio-economic status. In a country with a maternal mortality rate of 512 pregnancy-related deaths per 100,000 live births, only 12% of births in the poorest households were assisted by skilled birth attendants, compared to 87% in the richest households (3). Beyond marked disparities based on socioeconomic status, inequalities in access and use of maternal healthcare exist across geopolitical zones in Nigeria. Women in Northern Nigeria are less likely than women in Southern Nigeria to use skilled maternal healthcare services (3). Furthermore, the rural-urban divide brought about by unequal distribution of health resources and services among other reasons has manifested in the lower use of skilled maternal care services in rural areas compared to urban areas. Women's access to healthcare is moderated by several factors including the high cost of maternal services, the social and cultural mismatch between compassionate traditional care and often disrespectful care at skilled health facilities, and distance to healthcare facilities compounded by poor road and transportation infrastructure (4).

There is a growing consensus that the healthcare system in Nigeria faces enormous challenges to mitigate the aforementioned issues thereby necessitating the need for digital technologies to supplement, but not replace, existing maternal healthcare services. It is no surprise that Nigeria is prioritizing mobile health technologies to increase equitable access to and uptake of skilled maternal healthcare services and ultimately improve maternal health outcomes (5). However, mHealth is not a panacea, its potential to promote health equity can only be achieved if it is undertaken in a contextually relevant manner where it retrenches rather than exacerbate inequities (4). To better understand how mHealth can exacerbate inequalities including that involving gender, the next section discusses gender biases in mHealth.

### Understanding gender inequalities in mHealth

Per the sustainable development goal of ensuring healthy lives and wellbeing for all at all ages, digital health is projected to extend universal health coverage to 1 billion more people globally and ensure that more people enjoy better health and wellbeing by 2030 (2, 6). Universal health coverage can contribute to improving access to health services, particularly maternal healthcare services for the most vulnerable and underserved. However, celebrations of the anticipated progress of digital health are tempered with gender blind spots that pose a barrier to sustainable progress. Gender-based digital exclusions continue to push women and girls to the margins of society. Across low and middle-income countries, women are 15% less likely than men to own a mobile phone (7). In Nigeria specifically where mobile phone ownership ranges from 95% of the population in urban areas to 82% in rural areas, 14% more men than women in urban areas have ownership of a mobile phone while 35% more men than women are mobile phone owners in rural areas (3). This presents a significant challenge to women's ability to benefit from mHealth interventions. It must be understood that mHealth programs take place within complex social-cultural, economic and political settings and while they can transform aspects of these settings and have diverse impacts on equitable access to healthcare, their use and reach would largely reflect the wider socio-cultural, economic or political context (8). For instance, if the wider socio-cultural context allows gender-related constraints to prevent meaningful participation with digital technologies, it will be reflected in who can use and benefit from mHealth. Against this backdrop, a gender analysis is necessary when planning and prioritizing mHealth interventions.

To be effective, a gender analysis must go beyond the binary and heteronormative differences between girls and boys, men and women, and acknowledge the socially constructed nature of gender and the experiences within and among different groups of women, men, gender diverse people or gender non-conforming people (1, 9–11). Biases that perpetuate gender inequalities are rooted in unequal power relations between genders and how power is constituted to determine access to resources, division of labor and decision-making authority (10). Women's access to maternal healthcare is hindered when they lack access to resources, have limited decision-making power, face the burden of unfair division of labor, and bear the brunt of unfavorable social norms (10, 12, 13). Varied experiences of gender intersect with social positions, creating hierarchies of privilege and power that structure people's lives. Individuals can experience multiple oppressions at various points of intersection (14, 15). For instance, a study found that across sub-Saharan Africa, mobile health interventions demonstrated exclusionary practices at various intersections of women's identities (16). Not only were women excluded from participating in digital health programs based on mobile phone ownership, but they were also digitally excluded due to disabilities or low literacy levels. Ironically, these are often women who already have poor access to maternal healthcare services and are at risk of poor maternal health outcomes (16, 17).

Studies have presented a compelling case for the importance of understanding existing gender dynamics in mHealth interventions for maternal health (8). Programs that aim to address demand-side issues such as inadequate health knowledge and lack of affordable transport to healthcare facilities would often require that beneficiaries have access to mobile phones but without properly acknowledging the gender gap in mobile phone ownership, these programs can miss the intended beneficiaries. Furthermore, the lack of a gender and equity focus in the design of mHealth programs runs the risk of exacerbating inequality in access to health services and even enabling men's appropriation of women-centered programs (7, 8). A mHealth program aimed at improving women's knowledge of HIV/AIDS saw higher participation from men than women. The study concluded that due to gender gaps in mobile phone ownership, it unintentionally reinforced gaps in access to health information by providing more services to men than women, and the project was deemed unsuccessful (18). Additionally, while mHealth interventions can provide timely information on pregnancy and child health practices, they may threaten familial and community relationships if recommended practices conflict with the norm or if sensitive medical information is exposed. This is of particular importance when phone sharing patterns are taken into consideration. A study in Kenya indicated that women were more likely than men to share their phones with household and non-family members (19). Similarly, an SMS-based program designed to improve ART adherence among pregnant women with HIV reported that 84% of participants shared their phones (20). Participants expressed concern over the risk of exposing their HIV status to family and non-family members due to the program's overt HIV-related text message content. Taken together, these examples show that the success and continuous use of mHealth programs for maternal health are contingent on recognizing the implications of women's participation in these programs from a gender perspective.

The relational nature of gender and associated ills that arise due to gender inequalities is interwoven through the research reported above and through much of what is reported in this paper. Infusing a gender perspective in mHealth does not mean working with women in isolation but involves working across silos to engage women's broader social networks including men, community leaders, and decision-makers to bring about transformational change in gender relationships and sustain the benefits of mHealth. Addressing issues related to gender with women alone carries the risk of reinforcing their exclusion from the digital space and can worsen negative unintended consequences of participating in mHealth (8). This is because men and community elders are more likely to be enforcers of gender power relations and have the potential to positively advance gender relations for the benefit of those marginalized by gender power relations who are often women (10, 21). Yet, studies rarely engage these diverse perspectives in addressing issues and seeking equitable and sustainable solutions. Furthermore, while mHealth programs hold promise for extending maternal health services to underserved populations, they are rarely assessed on their differential impacts along gender lines. A vast majority of mHealth programs for maternal healthcare in Nigeria report on the feasibility or acceptability of mHealth programs (22–24), but research assessing the gender dimensions of mHealth interventions in communities remains scarce.

This study explores gender dimensions of a mHealth intervention for maternal healthcare in Edo State, Nigeria by capturing the unique perspectives of women who are the primary beneficiaries of the intervention, their spouses who are all men, and community leaders who oversaw the implementation and delivery of the mHealth intervention. The ensuing discussion is guided by three major questions (8): Do women have the resources to access and use the mHealth program? How does the use of the mHealth intervention impact gender dynamics and relationships? What markers of social inequality impact women's access to mHealth programs?

## Study setting

This study assesses the impact of the Text4Life program, a mHealth intervention aimed at increasing women's use of healthcare facilities for maternal, newborn and child health in rural Edo, within the context of the Innovating for Maternal and Child Health in Africa (IMCHA) project (25). This study was conducted in Etsako East and Esan South East; they are two local government areas (LGAs) of Edo state. Edo state is one of Nigeria's 36 states and home to eight million people, half of which live in rural areas (26). The two LGAs Etsako East and Esan South East are in the Northern part of the State and are predominantly rural. Participants were recruited from two communities within the two LGAs, Okpekpe from the Etsako East LGA and Ewatto from the Esan South East LGA. Both communities have two primary healthcare centers each covering 20 villages. The study sites are characterized by weak health infrastructure and high rates of maternal mortality. Furthermore, primary healthcare facilities serve as their main source of healthcare with no secondary or tertiary healthcare facilities located within any of the communities. The study sites were chosen based on the aforementioned characteristics.

### Text4Life: A description of the intervention

In 2017, the Women's Health Action Research Center (WHARC), a research-based non-governmental, non-profit organization in Nigeria in conjunction with the University of Ottawa, Canada, conducted formative research with the women, men, community elders, and policymakers in Etsako East and Esan South East. The studies identified challenges related to transportation, cost, and availability of healthcare providers as major deterrents to the use of primary healthcare centers in rural Edo communities, particularly for maternal healthcare (27–30). The mission of WHARC is to advance women's health in Africa through research and evidence-based advocacy. The center is located in Benin, Edo state Nigeria. Solutions proffered by the community led to the creation and implementation of the Text4Life Program from June 2019-December 2020. The mHealth program implementation was overseen by the Ward Development Committee (WDC) in each Local Government Area. The WDC constitutes select community members and a chairperson who serves as a bridge between health facilities and their communities. WDCs monitor the quality of service delivery to the community and enhance community involvement in health by harnesses community resources (human, financial, material) to sustain quality healthcare delivery (31).

Text4Life uses a web user interface created for the project by Textit^®^ to enable instant reporting of pregnancy-related events and timely notification of health facilities. The short message service (SMS) system-based technology enables dual communication between pregnant women and healthcare providers. It functions as an alert system and supports the documentation of pregnancies in the community. Pregnant women also receive health messages on topics around preconception, pregnancy, childbirth, and the postnatal period. To use this mHealth service, women need to have access to a mobile phone including shared phones, pay a registration fee of 2000 Naira (equivalent to $6.00) to join a community health fund, and provide their contact information including name, residential address, telephone number, and telephone numbers of the next of kin. Through the Text4Life platform, pregnant women are able to request swift transportation to primary health facilities during obstetric emergencies. If a pregnant woman is in distress, she can send a keyword to a registered number *via* SMS signaling distress. The message, in form of a dual SMS, is relayed to both the health facility and the WDC chairperson. The WDC chairperson is responsible for dispatching a community driver to pick up the woman in distress while the health workers prepare to receive the woman at the health facility. Women registered in this program do not pay additional fees for antenatal care, childbirth, postpartum and referral services at their primary health facilities. To enhance women's participation in the Text4ife program, WHARC loaned out phones to women through the health facilities for the duration of their pregnancies. Okpekpe community received 3 phones while Ewatto community received 10 phones.

**Figure 1:**
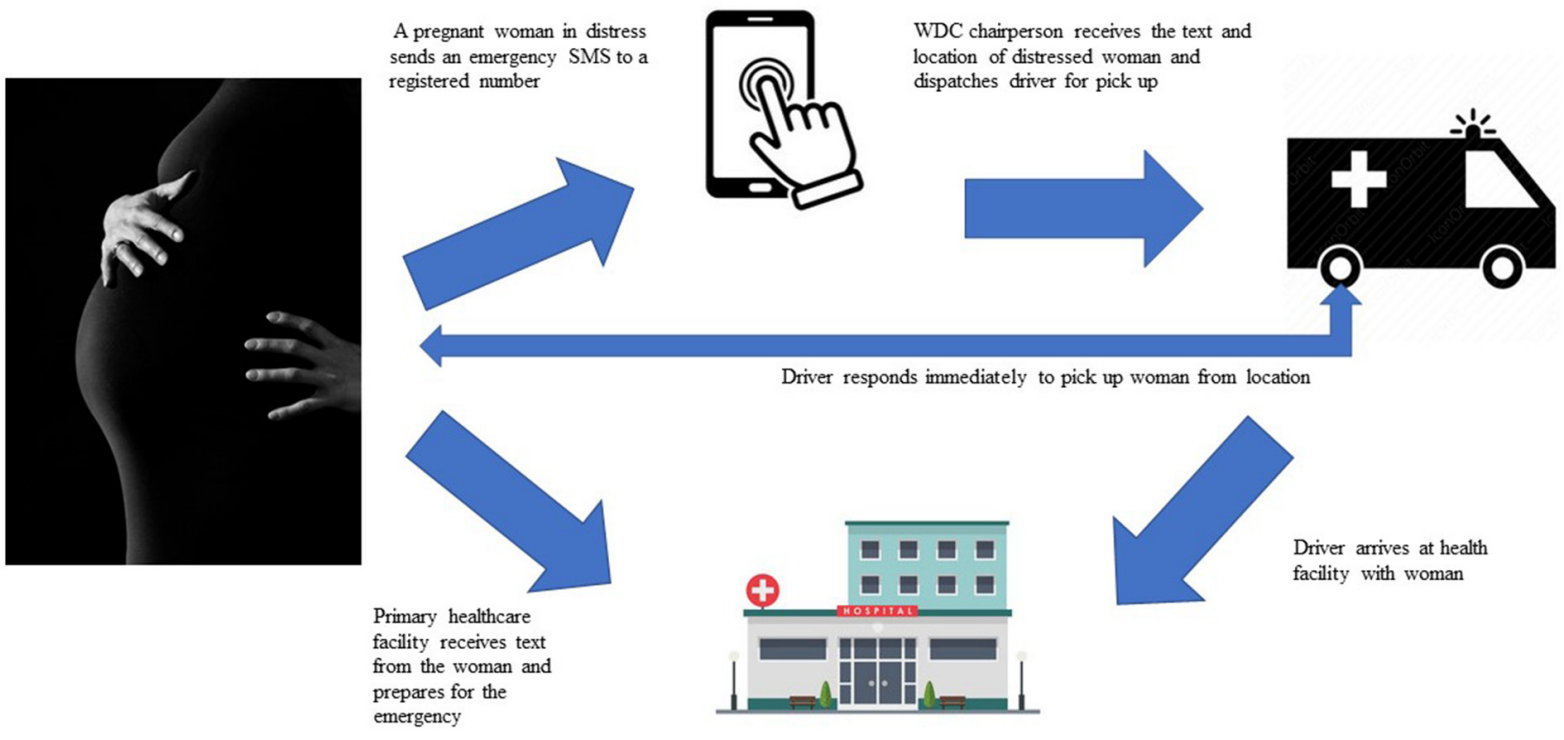
Overview of the Text4Life program.

See Figure 1 for an overview of the Text4Life program design.

## Materials and methods

### Study design

This is a cross-sectional qualitative study of women who registered on the Text4Life platform, their spouses, and the WDC chairpersons in both communities. This study presents findings from focus group discussions (FGDs) and in-depth interviews and takes a subjectivist inductive approach to the use of theory (32). This approach follows a bottom-up process of working from the data up to conceptualizations and rests on the assumptions of individuals' socially and experientially constructed realities and the researchers' responsibility to acknowledge these realities and explore meanings constructed by individuals. By acknowledging that reality is subjective and varies from person to person and by centering the participants as holders of knowledge, this study draws on constructivist ontology and epistemology (33).

### Theoretical approach

**Figure 2:**
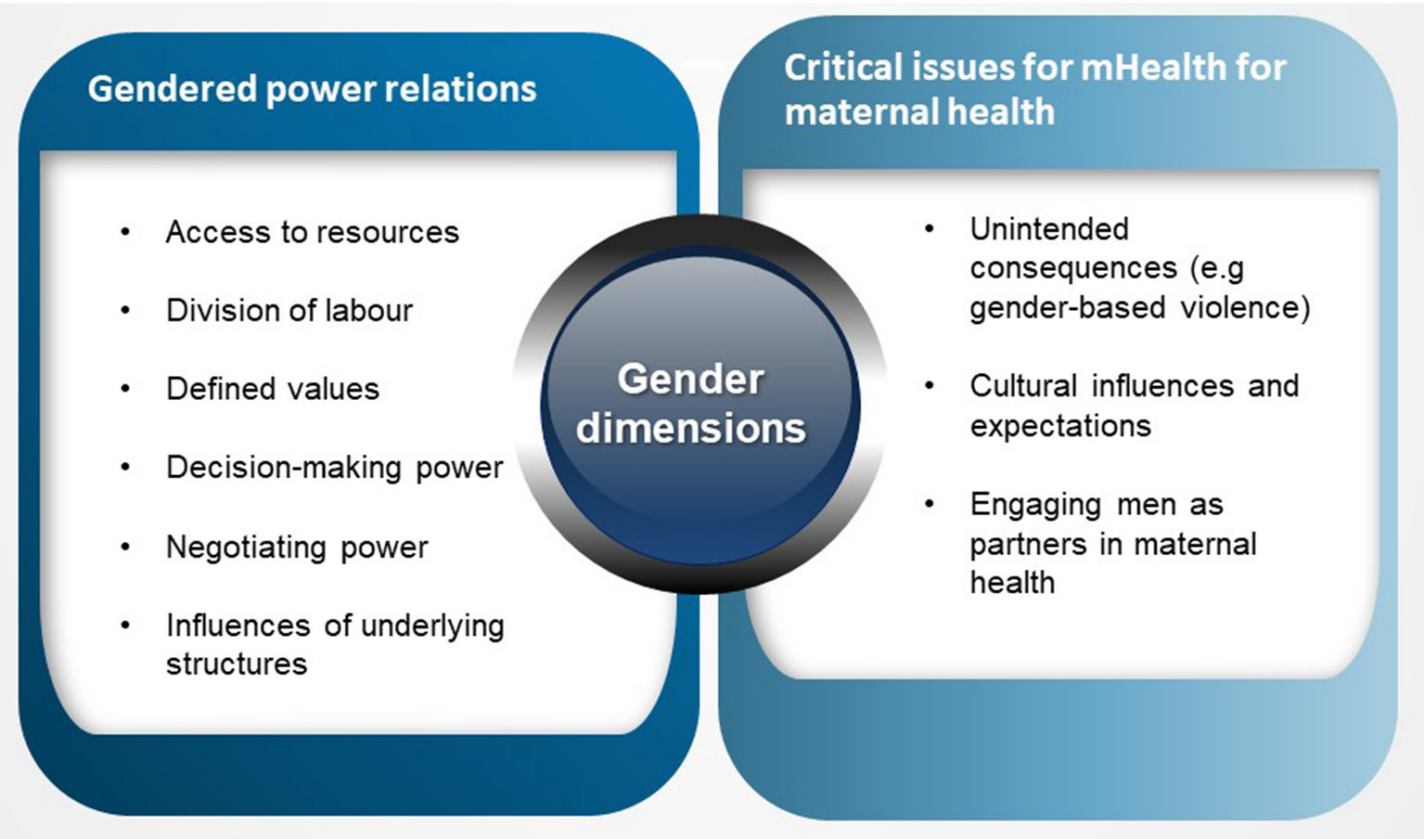
Operationalizing theoretical frameworks exploring gender dimensions in health interventions.

Guiding the study was an understanding of gender within the context of social determinants of health. Beyond the biological and physiological health needs, the social construction of gender which varies across stratifiers such as age groups, cultures, and socioeconomic class generates specific health situations and conditions for individuals (34). Attaining equity in health means recognizing the different health needs and identifying drivers of health inequities. Gender has been identified as a driver of inequity in access to healthcare in various frameworks. The WHO's social determinants of health tool identified key factors that impact women's health including gender roles, degree of access to and control over resources needed to safeguard health, aptitudes and skills (34). Women's roles and skills are judged to be less significant than roles and skills associated with masculine spheres. Morgan et al.'s (10) gender framework in health systems expands on the social determinants of health tool and argues that gender power relations are constituted and negotiated through access to resources, division of labor and their health implications, social norms and decision-making power (10). All of these factors are arguably drivers of inequity in health. Furthermore, Deshmukh and Mechael's (21) framework on gender in mHealth within maternal, new-born and child health argues that critical components to the success of mHealth interventions are gender-related issues including an understanding of unintended consequences of mHealth such as gender-based violence, a focus on women's empowerment, and engaging men and other relevant stakeholders that inform and shape gender relations and practices (21). These frameworks operationalized gendered approaches to health research (Figure 2). They address the research questions posed in this study and therefore provide a meaningful guide and basis for interpreting the data.

### Participants recruitment

As at 2019, there were 74 women registered for the Text4Life intervention from both Okekpe and Ewatto communities. While the sample size determination for this study was adaptive and emergent, this study relied on sample size guidelines for focus groups by Guest et al. (35) who observed that almost all themes emerge within three to six groups depending on the scope of the research (35). Furthermore, this study followed recommendations by Fush and Ness who argue in favor of the richness and depth of the data (36). The goal for this study was to stress the uniqueness of experiences across communities by obtaining thick and rich information through focus group discussions with participants in each study site.

This study used criterion-based purposive sampling to recruit a total of 66 participants: 39 women, 25 men and two WDC chairpersons. Women were target participants if they registered and/or used the Text4Life program. The next target group was men whose spouses registered and/or used the Text4Life program. Finally, WDC chairpersons who were key in the implementation of the program were the last target group of participants. Participants were recruited from the Text4Life database containing their names and phone numbers which were collected upon registration. Participants consented to have their contact information collected and stored on the Text4Life database by WHARC for research purposes. This database is not publicly available, however, the authors received authorization from WHARC and community members through the WDC chairpersons to access the database for research purposes only.

Two local research assistants conducted data collection. The research assistants, one man and one woman each possessing a bachelor's degree or higher level of education, are experienced in qualitative method data collection. The research assistants made telephone calls to women on the Text4Life database to solicit their participation. They adapted a telephone script for participant recruitment developed by OU. Eligible participants were women between the ages of 15 and 45 years old who used the Text4Life program during their most recent pregnancy. The research assistants explained to participants that the study aimed to understand their experiences with the Text4Life program. Research assistants followed up with men and introduced the study and solicited their consent to participate. All women who were approached agreed to participate in the study but some of the men declined to participate. There were only two WDC chairpersons, they are both men, and each is from one of the local government areas. Both were contacted by research participants, and both agreed to participate.

### Data collection and procedure

The two research assistants conducted a total of eight focus group discussions (FGDs) with a total of 64 participants across the two communities between September 2021 and January 2022. Following a recommendation by Fusch and Ness, each group consisted of six to 10 participants (36). Groups were small enough for members to talk and share their opinions yet large enough to create a diverse group. Focus group discussions occurred at community squares. Discussions lasted between 45 and 60 min. Two in-depth interviews (IDI) with WDC chairpersons occurred at community centers and lasted for ~45 min. Research assistants and participants adhered to COVID-19 protective measures during the study which included wearing masks and social distancing. Sanitizers were also made available to participants. Each participant received 3,000 Naira ($10CAD) to cover transportation and refreshment expenses. Prior to participating in this study, participants provided their informed consent by signing a consent form. Research assistants facilitated FGDs in English or Pidgin English language.

Questions were translated verbally into Pidgin English language when necessary. Each FGD and interview was digitally recorded, and researchers took notes during the discussions. To allow for sex-desegregated data collection, FGDs were held separately for men and women. This was important to encourage open discussions of private experiences and minimize undesirable consequences such as spousal confrontation or abuse that may threaten the participants' or their family's stability (37). Women and men participants were further segmented by age with the intent to minimize the effects of age hierarchy as dictated by cultural norms which may hinder freedom of expression from younger participants (37).

Under the supervision of FO and LN, research assistants transcribed the recorded discussions and interviews. All parties are proficient in written and spoken Pidgin English and English languages. Both authors screened the transcripts for errors. Transcripts were reviewed and discussed with OU to ensure a shared concept of key terms. Participants' responses were either transcribed verbatim if they responded in English or translated if they responded in Pidgin English. OU who is also proficient in spoken and written Pidgin English and English languages translated the transcripts where necessary. Literal translation (word-by-word) was used to preserve participants' responses and provide readers with an understanding of the mentality of the participants (38). The process of translation involved back translation whereby data were translated from the source language (Pidgin English) into the target language (English) and back into the source to clarify any ambiguities or discrepancies (38). The findings are reported in English language. Any identifying information for participants was altered to protect their privacy, they are referred to simply as a man or woman participant instead.

#### Research instrument

Guides for focus group discussions and interviews were developed by OU. They were designed to capture contextual information related to the topic and elicit detailed discussions from women, men, and WDC chairpersons regarding their experiences with Text4Life. Questions covered topics related to their general perceptions of the Text4Life program, motivations for use, barriers, and facilitators to using the intervention, and perceived quality of the intervention (39, 40). Questions were carefully crafted to include neutral, non-biased, and non-leading questions to avoid influencing participant responses. Guides were modified where appropriate to suit the local language, literacy levels and cultural interpretations.

### Data analysis

The transcripts were exported to NVivo 1.6 and OU led the data coding process which was heavily inductive. This study used a conventional approach to thematic analysis to explore responses from FGDs and IDIs (33). First, the coder was immersed in the data and initiated coding with five apriori codes based on interview guides such as ‘cost for mHealth,” and “decision-making.” As coding progressed, existing codes were refined inductively with the discovery of newer codes such as “safety,” “status.” In this phase, the author allowed new insights to emerge. There was limited reliance on a pre-existing category, instead, codes and categories flowed from the data (41). Codes captured data relevant to the primary questions of this study as well as emergent themes that arose from the initial review. Upon identifying and defining codes, representative quotations from the transcript were assigned to different codes. Codes were reviewed and overlapping codes were further organized into categories. Themes were subsequently generated from categories that reflected a level of pattern in response or meaning. Table 1 indicates key themes, sub-themes and corresponding participants through whose perspectives subthemes and themes were constructed.

**Table 1:** Gender dimensions of the Text4Life intervention.

**Key themes**	**Sub-themes**	**Answers divided by participant**		
		**Women**	**Men**	**WDC chairpersons**
**Resources for mHealth use and access**	
1. Mobile Phone Ownership	• Gender gap in phone ownership: Women's limited phone ownership or independent control over phones	X	X	X
2. Mobile Phone Maintenance Challenges	• Connectivity challenges impacted the use of mHealth intervention	X	X	X
	• Fluctuating power supply impacted proper phone functions	X		X
	• The need for phones for women in the community	X	X	X
3. Cost Associated with mHealth	• Phones disbursed to beneficiaries for free.	X		X
	• Cost of mHealth program and pregnancy care offset by intervention.	X		X
4. mHealth Literacy	• Sufficient training provided on how to use Text4Life	X		X
**Markers of inequality**	
1. Women's Status	• Phone ownership as a means of changing women's status	X		
	• Maternal health under the purview of women		X	
2. Women's Decision to seek Pregnancy Care	• mHealth improved women's decision-making power	X	X	X
3. Women's Safety	• Women described as victims of crimes when seeking pregnancy care at night time	X		
**Gender relationships**	
1. Social Norms	• Women under scrutiny for interactions with other non-spouse men	X		X
2. Childbearing Intentions	• Women's reproductive life under men's control		X	
3. Men's Influence	• Success of the mHealth program contingent upon men's approval		X	X

### Ethics approval

A certificate of ethical approval was obtained from the University of Ottawa's Research Ethics Board—file number S-02-21-6573, and from Nigeria's National Health Research Ethics Committee (NHREC)—file number NHREC/01/01/2007–18/04/2017.

## Results

### Participant characteristics

Focus group participants ranged from 6 to 10 individuals per group. The median age for women was 26 years old and 45 years old for men, age categories are indicated in Table 2. The predominant level of education in these rural communities is primary education and the conventional occupation is farming.

**Table 2:** Study participants.

	**Participants**	**Contact type**	**Number of participants**	**Age range (median) in years**
1.	Ewatto women 1	FGD	6	34–39 (36)
2.	Ewatto women 2	FGD	8	25–30 (27)
3.	Okpekpe women 1	FGD	8	45–52 (45)
4.	Okpekpe women 2	FGD	10	40–47 (45)
5.	Okpekpe women 3	FGD	7	52–57 (54)
6.	Ewatto men	FGD	8	25–28 (26)
7.	Okpekpe men 1	FGD	8	20–25 (23)
8.	Okpekpe men 2	FGD	9	24–29 (26)

In this section, we explore the necessary resources for women's participation in the Text4Life program; we highlight the role of Text4Life in retrenching or magnifying social inequalities, and finally, we describe the impact of Text4Life on gender relationships and social norms in the community.

### Resources for mHealth use and access

#### Phone ownership

Participants generally described a gender gap in mobile phone ownership. Women who were the primary beneficiaries of the Text4Life program were less likely than men to own mobile phones. Women generally reported not owning or not having independent control over mobile phones. Lack of access to mobile phones constrained women's use of the mHealth program and deepened their dependency on their spouses. In the absence of their spouses who were often the primary owners and holders of mobile phones, participants reported that some women remained vulnerable in emergencies; some experienced delays in reaching care and others had home births without a skilled birth attendant. The implications of women's vulnerabilities were not lost on men as they acknowledged the risk women face without access to phones during emergencies, however, even in the face of precarity such as having a spouse who had neared her delivery date, men were not relinquishing their phones.

“*For most of the women in the village here, some of them do not have phones. If something happens, she will need to get to a nearby person to say help me call this number. The woman is already under stress, pain and everything. More phones should be provided so that in case of an emergency she can call. The phone will not be given to the women permanently, they will return the phone that will be in possession of the health care center.”* (IDI, WDC chairperson, Ewatto)

“*My wife, she has no phone to call me, so upon my return from the farm, I will be told congratulations. I will say what happened, and they will say my wife has delivered at home because I was not around*.” (FDG, Man participant, Ewatto)

Participants disclosed that women received phones on loan to participate in the Text4Life program, but it was not sufficient for all the women registered in the program. WDC chairpersons indicated that program participants from Okpekpe received three phones while women from Ewatto received 10 phones. Women were expected to use the phones for the duration of their pregnancy and return it after childbirth. Efforts to reduce the burden of limited phone ownership were acknowledged by recipients who emphasized that without the phones, they would not have been able to participate in the mHealth program at all. Men and WDC chairpersons raised concerns over the issue of ownership and feared that insufficient mobile phone ownership will hinder women's future use of the mHealth study.

“*The phone was free even if we don't have money, we could use it as we were not required to pay*.” (FGD, Woman participant, Ewatto)

“*Like I said, they only gave out three phones which were not adequate, not enough. The ability to possess a phone is important for pregnant women that's why I suggested that they can bring more phones*.” (IDI, WDC Chairperson, Okpekpe)

#### Phone connectivity and maintenance challenges

Women's mobile phone ownership issues were further compounded by network connectivity challenges and maintenance challenges. Most participants reported poor network coverage which impacted the use of the mHealth program. Poor and fluctuating supply of electricity was also highlighted as an issue for keeping phones charged to use Text4Life consistently. A few participants reported that women owned a backup power generator through which they are able to charge their phones. However, this was not a consistent response from participants. This indicates that beyond the gender gap in mobile phone ownership, wealthier participants were confronted with fewer challenges in using Text4Life than poorer participants.

“*My wife and I have phones, but we do not have steady power supply, but we use a generator. That is how we charge our phones*.” (FGD, Man participant, Ewatto).

“*Yes, you know in the village here, sometimes the network is rubbish, not only in my community but nationwide*.” (IDI, WDC chairperson, Ewatto)

#### Cost of mHealth

To access the Text4Life program, women were required to pay N2,000 ($6) to a community health fund. Many of them deemed this cost affordable. More so, their registration for the health fund gives them access to free pregnancy care at their primary health facility which encompassed antenatal care visits, childbirth and postpartum care. They also had free use of the mHealth program through which they received health-related messages and communicated one-on-one with healthcare staff at their primary healthcare facilities. They also received free transportation services to health facilities in emergencies. The regular cost of the services was considered exorbitant for a lot of women who cited the cost of healthcare as a barrier to their use of skilled pregnancy care in previous pregnancies. Women were happy with the current subsidized cost of healthcare as it allowed them access to skilled care even when they could not afford it. Men termed the cost structure of mHealth an achievement and a gift for many families. WDC chairpersons reported a significant uptick in the number of women using primary healthcare facilities since the implementation of the mHealth program.

“*It made pregnancy easier, the stress of worrying for lack of money was not there, things we needed were always available*.” (FGD, Woman participant, Ewatto)

“*Healthcare services became better with regards to the assistance with transportation, the free delivery fee and medical personnel being available to attend to us on the phone and in-person*.” (FGD, Woman participant, Okpekpe)

#### mHealth literacy

Women received training from WDC chairpersons at the point of registration to enable them to use the Text4Life program. WDC chairpersons confirmed their role in providing training to women. Women received additional training from healthcare providers at their health facilities during antenatal care. Women provided detailed descriptions of operating the Text4Life platform which was indicative of their understanding of how to use the theText4Life program on their phones and consequently, an indication of effective training.

“*We were trained on how to make it effective, how to use the tex4life, and when we came back to the village, we showed the women how to operate it on the phone whether it was the one provided by us or their own personal phones*.” (IDI, WDC chairperson, Okpekpe)

### Markers of inequality

#### Women's status

For some women, owning a phone extended beyond the need for healthcare but was also a means of changing their status. For them, participating in the Text4Life program meant taking charge of their health independent of their spouses. Some women took on extra responsibilities to earn money to purchase their own phones so as not to rely on their spouses. One woman noted:

“*During that period, I had to work hard in other to get myself a phone so I can be part of it, or else I will need to ask my husband for assistance*.” (FDG, Woman participant, Okpekpe)

#### Women's decision making

The mHealth program had implications for women's decision-making in health care. Participants acknowledged that men were the primary decision-makers in women's ability to seek care. Men attributed the role of decision-makers to themselves based on gender and cultural expectations of their roles as fathers. Women, however, demonstrated their influence over their healthcare-seeking decisions in various ways. For instance, some women reported that they tell their spouses where to take them for pregnancy healthcare. Other women actively sought care through the Text4Life service independent of their spouses. Chairpersons also recognized men's roles in determining women's use of skilled pregnancy care services. They stated that the mHealth program was an effective means of empowering women to decide to seek care independent of other factors including their spouse's permission or economic status.

“*Yes, with the awareness of it (Text4Life), every woman knows the importance of coming here. Women now whether their husband decides or not, whether he gives them money or not because they know it concerns their health, they find a way to come to the health center. There is hardly a woman now who has issues and refuses to come to the health center. Unlike before the program, the nurses are much busier now than before. The records are there, things have changed drastically*.” (IDI, WDC chairperson, Okpekpe)

#### Maternal health under the purview of women

Men's role in maternal health focused primarily on providing financial resources for maternal care. This was deemed an important role considering women's account of out-of-pocket expenditures for healthcare before the Text4Life program. However, some men indicated disinterest in women's health. They admitted to not often participating in community events related to maternal health and expressed surprise at the turnout of men for the focus group discussions. Some were generally unaware of the Text4Life program and its role in maternal healthcare in their communities. In one of the four focus groups with men, the majority of participants were unaware of the Text4Life program even though their spouses were registered on the platform. Some of the men argued that maternal healthcare is within the purview of women and did not require a man's involvement. Similarly, some women participants asserted that they are responsible for themselves during pregnancy therefore it was not surprising that some participated in the program unbeknownst to their spouses.

“*Me, my wife did not tell me anything about that…it's not everything that has to do with women that men do. Sometimes women will attend some of these programs but feel reluctant to tell their husbands. I see they are at fault*.” (FGD, Man participant, Okpekpe)

“*I am the one with the pregnancy, so I am the one to decide to go to the hospital*.” (FGD, Woman participant, Okpekpe)

#### Women's safety

Women's safety during pregnancy emerged as a key concern for participants, a concern they felt that the Text4Life program was addressing. Notions of safety emphasized protection and lack of harm during pregnancy and childbirth. Participants opined that women often faced precarious and unsafe conditions while reaching care, therefore notions of safety also meant protection from harm or crime while traveling for healthcare. Transportation assistance through the Text4Life program meant that women were not stranded on the roadside on their way to healthcare facilities, especially at night. Women unanimously agreed that they had safer childbirth experiences with the Text4Life program. Through the program, healthcare providers are alerted of an emergency before women's arrival and are prepared to offer prompt medical attention upon their arrival.

“*We are no longer stranded at night. There was a situation where a woman was stranded and delivered at home. She might have had bleeding issues or placenta issues. She now used Text4Life, and help came at midnight around 2 am. The Text4Life can help other people because in this area there are places that aren't accessible by cars and even riding a motorbike at night is sometimes problematic, but if we use this Text4Life, someone will come to our aid and save us and we now have a little safety and a successful life. We like this text4 life*.” (FGD, Woman participant, Ewatto)

### Gender relationships

#### Social norms

Participants' responses revealed a prevalent etiquette for women's interactions with men who were not their spouses. Men (non-spouse) and women incurred suspicions from their spouses and the community at large when they are seen together at night. Women indicated that their interactions with men drivers especially late at night left community members *wondering*. They felt it was important that drivers become well-known to the community. They also stated the importance of publicizing the program to the community to allay their suspicions. Similarly, WDC chairpersons describe the possibility of conflict arising from women's spouses due to women's interactions with them (WDC chairpersons) or with community drivers (men) at nighttime. They pre-empted conflicts by being cautious in their interactions and by raising awareness of their role in the Text4Life program.

“*Everybody in the community should know him as the taxi man and they should know that ordinarily, he can't come to the area because he lives far away from the woman in question. So, they must be wondering what made the man come out to that area at that time, then we can tell them that it is with the help of Text4Life that we were able to reach the taxi man who came and conveyed her to the health center*.” (FGD, Woman participant, Ewatto)

“*During that period, they had to know what I was doing, so they don't see me talking to a pregnant woman at midnight without her husband and think otherwise. I tell them I have no other connection with her other than to see that she delivers successfully*.” (IDI, WDC chairperson, Okpekpe)

#### Child-bearing intentions

The mHealth intervention evidently impacted men's construction of maternal bodies as sites through which women's reproduction could be regulated. Men participants extolled the benefits of Text4Life in ensuring safe pregnancies for women so much so that it heightened their intentions to have more children. Conversely, women praised the positive impacts of Text4Life in optimizing maternal care but did not give any indication of its influence on their childbearing intentions. For some men, safe maternal health as a result of the Text4Life program meant prolonging women's reproductive years, a decision seemingly taken without women's input. This signified men's control over women's reproductive lives.

“*Personally, the program is very good. In fact, I still want my wife to give birth twice again because you can just call at any time. So even at 60 years, she can still give birth*.” (FGD, Man participant, Ewatto)

#### Men's approval of mHealth

A latent but important theme was an indication of men's influence in sustaining the intervention but only if they approved of it. The men participants indicated their approval of the program and permitted the program to continue in their communities. Furthermore, the success of the program seemed contingent on Chairpersons' efforts such as supporting the program using their personal resources in addition to those provided by the program. WDC chairpersons also indicated that some men who were not designated drivers also assisted with transportation.

“*We sit here and welcome you to this place. This program (Text4Life) is very good. It has changed some of us. This time around you can stay*.” (FGD, Man participant, Ewatto)

“*As the WDC chairman, which I still am, I have played my part, I have been playing my part especially when WHARC came here for research in our community. I played all the required roles in assisting them acting as an intermediary between the community, the women in particular. During pregnancies and delivery for the women, I liaise with the doctor and nurses and also ensure that the women follow instructions given to them by the nurses. With the transport system I ensure that the drivers were timely when needed I make sure in the absence of the driver, I use my car to help women get to health centers for delivery or serious cases when need be*.” (IDI, WDC chairperson, Okpekpe)

## Discussion

As communities across sub-Saharan Africa continue to leverage the use of mHealth for maternal health, this study provides insight into the gender implications of women's use of mHealth technologies. The study will influence how mHealth interventions for women as end-users are designed and deployed. Specifically, this study focused on Text4Life, a mHealth program aimed at improving women's access to skilled pregnancy care in rural Edo, Nigeria. We explored women's access to resources to participate in the study including mobile phone ownership and the financial cost of the mHealth program. We commented on markers of social inequality that were either amplified or redressed by the mHealth program. Finally, we discussed the impact of Text4Life on gender relationships in the community. In general, we observed that while mHealth programs are helpful to women in many ways, they are not enough on their own to undo entrenched systems of power through which men control women's access to resources, women's reproductive lives, and social lives.

All digital health programs occur in distinct social, economic and political contexts. It is not surprising therefore that women's use and access to mHealth as described by participants reflect, to a large extent, the wider context within which such technologies are produced and used. Women have less access to phones than men and are less likely to participate in digital spaces. They are often excluded from the benefits of mHealth especially when programs are designed without any regard for gender, age, ethnicity or disability. Our study corroborates findings across sub-Saharan Africa that have long-established the gender disparities in mobile phone ownership (42, 43). For instance, despite the high presence of digital health tools and their potential in redressing maternal mortality in Southern African Development Countries, rates of maternal mortality in this region remain high (44). Reasons for this were attributed to the gender disparities in access to digital health tools. Similarly, our study shows that women were more likely than men to report shared use of phones. Men and women acknowledged this as an issue and a barrier to using the mHealth program. However, as our results demonstrate, men were not relinquishing their phones even in the face of an impending emergency.

Evidence from our study shows that the provision of free phones for program beneficiaries (pregnant women), though insufficient for all participants, was a promising means of ensuring their participation in the program. Similar findings were reported in a rural community in Southern Nigeria where pregnant women received free phones to participate in a mHealth intervention. The study reported that recipients of the phones found them helpful, and their use of primary healthcare facilities improved (45). However, other studies illustrated that simply providing free or subsidized phones for beneficiaries of mHealth programs in underserved communities does not guarantee their use (46). An initiative in Kenya and Rwanda saw limited phone usage even with the provision of phones for women. Limited phone usage was attributed to ongoing costs to maintain phones, limited digital literacy, and social norms that discourage women's use of phones (46). Similar issues were highlighted as barriers to women's use of Text4Life in our study. Efforts to avail phones to women must be carried out alongside interventions that address: the ongoing costs of phones, digital literacy and negative consequences of phone ownership at the household level. Network coverage especially in rural areas is imperative to foster a more inclusive digital world. A report on Nigeria's digital health landscape acknowledges infrastructural issues such as network coverage, but more investments are needed to enhance network coverage, especially in rural areas (5). Furthermore, gender-oriented targets for digital technology infrastructure (e.g., network coverage, affordability of technologies) should be at the core of efforts to enhance Nigeria's digital health landscape.

Participants reported that registration to access the mHealth program cost N2,000 and it gave women the added benefit of access to pregnancy care including antenatal, childbirth and postpartum check-up at no additional cost. The consensus was that it was affordable and a fraction of the cost of healthcare in these communities. Previous studies conducted in these communities indicated that women pay ~N10,000 for pregnancy care, with childbirth alone costing N2,000 (27, 47). It is noteworthy that during consultations with various stakeholders in maternal health including women, policymakers, clinical managers and community elders, they advocated for free or subsidized healthcare to increase women's use of primary healthcare facilities in their communities. Our study affirmed that subsidized user fees improved women's use of the mHealth program and health facilities. This is of particular importance because, in the formative studies, it was observed that women's dependency on their spouses' authorization before seeking care was deepened by high levels of poverty. Furthermore, our study shows that women's autonomy and ability to decide their own care without permission or coercion were expanded by the mHealth program. Similar to our study, other studies across sub-Saharan Africa confirm that subsidized cost of pregnancy care improves women's use of skilled pregnancy care (48).

WDC chairpersons opined that as a result of the mHealth program, women were no longer reliant on their spouse's authorization before seeking healthcare. Some women shared the same opinion and demonstrated their independence by partaking in the mHealth program unbeknownst to their spouses. There is a likelihood that this is a strategy to avoid conflict. While this was not explored extensively in our study, a plausible explanation was given in a Kenyan study where participants cited a lack of trust and fear of violence for women who covertly participated in a mHealth program for maternal health (49). It is noteworthy that women's sole decision-making does not necessarily improve with the provision of free or subsidized healthcare. Findings from Burkina Faso stated that even in the context of free maternal healthcare, husbands' authorization was still necessary for using maternal healthcare and women were deemed stubborn or domineering if they ventured on healthcare decisions alone (50). Interestingly, the study revealed that joint decision-making among couples was the most acceptable health-seeking behavior.

The literature on men's involvement in maternal healthcare in various African contexts highlights how unequal social status and power relations begin at the household level (51, 52). Due to gender inequality, women's sexuality can become a matter of compulsion and not a choice, therefore in cases of early marriage, availability of sex, contraceptive use, number of children or even consensual marriage, men, not women, are primarily in control of those choices (51). Our findings showed that despite men being referred to as the major decision-maker in seeking healthcare for pregnancy care, maternal health was generally under the purview of women and some men participants demonstrated limited knowledge and interest in maternal health. This is important as it implies that men are likely to have inadequate knowledge of medical threats or danger signs during pregnancy which inevitably leads to deprioritizing adequate maternal healthcare. Similar findings recognize men's limited involvement in maternal health as a barrier to women's access to skilled care (53). In contrast, studies in Ghana and Sierra Leone re-examined men's involvement in maternal health and argued that men's involvement occurs in contextual and culturally gendered ways such as financial responsibilities and emotional care. Women did not welcome male partner involvement in maternal health beyond the provision of money for healthcare and transportation costs (54, 55).

Our study shows that the Text4Life program increased women's feeling of safety during pregnancy. Safety was expressed both as hospital-based births free of obstetric danger or properly managed complications; and physical safety from harm and crime while traveling to the healthcare facility. Safety is a primary aim of various mHealth programs across sub-Saharan Africa although they often focus on clinical safety (56). Similar to our study that defines safety in terms of enhancing safety from crime during travel, the definition of safety in mHealth has gone beyond clinical safety and encompasses outcomes such as confidentiality, privacy of patient information, and emotionally and socially safe mHealth service (57, 58). Interestingly, in our study, women's emphasis on a safe pregnancy sharply contrasted with men's emphasis on continual childbearing as a result of safe pregnancies. While Text4Life enabled safer pregnancies, it exacerbated opportunities for men's control over women's reproductive lives. Corroborating our findings is a multi-country analysis of unintended pregnancies across sub-Saharan Africa. The study shows that women's lack of control or participation in family size decisions increases the risk of unintended and short-interval pregnancies which consequently predisposes women to several risk factors such as unsafe abortion, higher chances of pregnancy complications, maternal deaths, and mental health issues (59).

Our study also examined changes in gender relationships due to the mHealth program. We acknowledge that this topic traverses findings already reported. We observed that while mHealth expanded women's autonomy in healthcare decisions and healthcare seeking, it seemed to pose a threat to men spouses whose notions of gender relationships have been conditioned by patriarchal norms that seek to maintain control over women. Women's participation in the mHealth program created the possibility of conflict in their relationship with their spouses and the community as a whole. This was also an issue that impacted WDC chairpersons who feared confrontation from women's spouses. Evidence from a systematic review on mHealth interventions and gender relations affirms this study by showing that women's risk of experiencing domestic disputes increases during their participation in mHealth programs (60). Reasons for this were attributed to women's enhanced decision-making or autonomy which threatened men who have been conditioned to maintain control over women.

Corroborating other research involving men across sub-Saharan Africa (61), our findings show that men wield power in the sustainability of the mHealth program. In their role as WDC chairpersons or as spouses, if they approved of the program, they provided the necessary human resources to contribute to the program's success. Formative studies conducted in the same community with elders (who are predominantly men) showed that they voluntarily offered lands as sites for new hospital buildings (27). It is important to note that under most customary laws in a Southern Nigerian context, land is communal, meaning that it belongs to families, villages or communities but not individuals, however, primary leadership over land is conferred on men more than it is on women, therefore, they have higher access to and control of resources including financial and natural resources (62).

Implications of our findings on men's influence are 2-fold; first, it highlights the importance of men's involvement in maternal health in a gender transformative manner that seeks to promote respectful and equal relationships that change harmful gender norms. Emerging evidence from the literature shows that transformative change is possible but requires a shift from a fixed notion of masculinity and attention to specific contexts (49). Second, it is important to explore means of elevating women's leadership in overseeing the design and implementation of maternal health programs within our study communities. This gives women a voice in determining programs they need but also enables them to take ownership and ensures the sustainability of programs.

### Study strengths and limitations

This study draws on the diverse perspectives of women, their men spouses and WDC chairpersons and enables a holistic consideration of gender dimensions in the study. Variations and similarities in perspectives brought issues around gender dynamics and power relationships in mHealth programs to great relief in ways that may be less apparent with only one group of participants. Additionally, this study was sensitive to gender dynamics and included those considerations as described in the methods section. Focus group discussions were disaggregated to ensure women-only and men-only groups. The study had a man and woman research assistants conducting focus group discussions and interviews to cater to participants' preferences, improve their comfort level and build rapport.

The strengths of the study notwithstanding, our work presented here also necessitated critical reflection and acknowledgment of its limitations. The degree to which probing was employed as a technique by the research assistants may have influenced the depth of responses generated by participants on some topics. However, the use of research assistants who could converse in the local languages undoubtedly impacted the dynamics between participants and researchers and improved rapport. Furthermore, the study did not gather information on participants' characteristics in great detail, nor does it report on other contextual situations that could have impacted participation in the Text4Life program such as marital status including polygamous families, female-headed households, and ethnic differences, among others. We have addressed this by citing relevant formative research undertaken within our study communities as part of the larger study. Finally, this study did not examine the aftermath of men's knowledge of the usefulness of mobile phones for their spouses, there is a possibility that this will influence mobile phone ownership among women.

### Future research

Our findings and discussion show that if underlying gender dynamics that impact maternal health are to be addressed, gender must be integrated into mobile health interventions for maternal healthcare early on in the intervention process. Ongoing work in these communities already engages with those who enforce gender norms in the community such as community elders and men. Future research can investigate how to build on best practices while findings creative ways to challenge gender norms that lead to inequalities and consequently impacts maternal health. Future research can investigate women's leadership in digital health spaces, particularly in Nigeria's rural communities.

## Conclusion

As communities across sub-Saharan Africa continue to leverage the use of mHealth for maternal health, this study provides insight into the gender implications of women's use of mHealth technologies. Through focus group discussions with women and men, and interviews with ward development committee chairpersons, our study highlights the many benefits of Text4Life but emphasizes that failure to address underlying gender inequalities and unintended consequences of mobile health programs limits its benefits and ultimately its sustainability. While women did have some of the necessary resources to access and use the program, they were insufficient and derailed their participation in the Text4Life program. The program enhanced women's status and decision-making capacity but with men positioned as heads of households and major decision-makers in maternal healthcare seeking, there remained the possibility of deprioritizing maternal healthcare at the household level. Text4Life expanded women's sphere of autonomy and interactions but that came with the possibility of exacerbating conflict within the family and community. While Text4Life prioritized women's safety in various contexts, it entrenched systems of power that allow men's control over women's reproductive lives. The evidence provided by our study demonstrates the need to integrate gender into digital health programs because, in addition to having limited resources that constrain women's participation, they are navigating wider structures of patriarchy where gender inequality is reinforced. This will help inform digital health policy that promotes and nurtures gender equality at all stages of a program's development.

## Data availability statement

The raw data supporting the conclusions of this article will be made available by the authors, without undue reservation.

## Ethics statement

The studies involving human participants were reviewed and approved by the University of Ottawa's Research Ethics Board—file number S-02-21-6573 and by the National Health Research Ethics Committee (NHREC)—file number NHREC/01/01/2007–18/04/2017. The patients/participants provided their written informed consent to participate in this study.

## Author contributions

OU conceptualized the study, coded and analyzed the data, and prepared the manuscript with input from SY, FO, and LN. FO and LN coordinated and directed data collection. All authors read and approved the final manuscript.

## Conflict of interest

FO is the founder of the Women's Health Action Research Center WHARC, the organization that implemented the Text4Life study. The remaining authors declare that the research was conducted in the absence of any commercial or financial relationships that could be construed as a potential conflict of interest. The handling editor BE declared a past co-authorship with the authors SY and FO.

## Publisher's note

All claims expressed in this article are solely those of the authors and do not necessarily represent those of their affiliated organizations, or those of the publisher, the editors and the reviewers. Any product that may be evaluated in this article, or claim that may be made by its manufacturer, is not guaranteed or endorsed by the publisher.

## Author disclaimer

All views expressed in this paper are of the authors only.
